# A Descriptive Study of the Types and Survival Patterns of Saudi Patients with Multiple Primary Solid Malignancies: A 30-Year Tertiary Care Center Experience

**DOI:** 10.3390/curroncol29070393

**Published:** 2022-07-13

**Authors:** Moustafa S. Alhamadh, Rakan B. Alanazi, Sultan T. Algarni, Ahmed Abdullah R. Alhuntushi, Mohammed Qasim Alshehri, Yusra Sajid Chachar, Mohammad Alkaiyat, Fouad Sabatin

**Affiliations:** 1 College of Medicine, King Saud Bin Abdulaziz University for Health Sciences (KSAU-HS), Ministry of the National Guard—Health Affairs, Riyadh 14611, Saudi Arabia; rbfa19@gmail.com (R.B.A.); sultantheepan@gmail.com (S.T.A.); ahmed.alhantoshi@gmail.com (A.A.R.A.); m.q.112@hotmail.com (M.Q.A.); 2 King Abdullah International Medical Research Center, Ministry of the National Guard—Health Affairs, Riyadh 11481, Saudi Arabia; chachary@ksau-hs.edu.sa (Y.S.C.); alkaiyatmo@gmail.com (M.A.); fsabatinmd@gmail.com (F.S.); 3 College of Sciences and Health Professions, King Saud Bin Abdulaziz University for Health Sciences (KSAU-HS), Ministry of the National Guard—Health Affairs, Riyadh 14611, Saudi Arabia; 4 Department of Medical Oncology, King Abdulaziz Medical City, Ministry of the National Guard—Health Affairs, Riyadh 12713, Saudi Arabia

**Keywords:** multiple primary malignancies, multiple primaries, synchronous malignancies, metachronous malignancies, second primary

## Abstract

Background and Objective: Cancer survival has improved significantly, which reflects the achievements in screening, diagnosis, and treatment. As a consequence, multiple primary malignancies are diagnosed more frequently, with an incidence ranging from 0.52–11.7%. The types of malignancy that coexist and survival patterns vary notably in different countries and geographical areas. Due to the limited literature in Saudi Arabia, a baseline of prevalent malignancy combinations and their survival patterns would support early detection and disease management. Method: This was a retrospective descriptive study conducted from 1993–2022 at King Abdulaziz Medical City, Department of Medical Oncology, Riyadh, Saudi Arabia. Patients with at least two biopsy-proven solid malignancies were included. Patients with hematological malignancies, missing data, or an uncertain or indecisive pathology report were excluded. Result: In total, 321 patients were analyzed. More than half (57.3%) of the patients were female. A third (33%) of the cases were synchronous, and 67% were metachronous. The most frequent site of the first primary malignancy was breast cancer, followed by colorectal, skin, and thyroid cancers. The most frequent site of the second primary malignancy was colorectal cancer, followed by thyroid, breast, and liver cancers. Only 4% of the cases had a third primary malignancy, with colorectal and appendiceal cancers being the most frequent. The most frequently observed histopathology in the synchronous and metachronous malignancies was adenocarcinoma. Breast–colorectal, breast–thyroid, and kidney–colorectal were the most frequently observed malignancy combinations. Conclusion: The current study offers a baseline of multiple primary malignancies in Saudi Arabia and provides supporting evidence that the pattern of multiple primary malignancies varies among different countries and ethnicities. The possibility of developing another primary malignancy should be considered when treating and monitoring cancer patients.

## 1. Introduction

Cancer is the second most frequent cause of mortality, and a major burden that is increasing globally [1,2]. In 2008, globally, 12.7 million new cancer cases and 7.6 million cancer fatalities were estimated [2]. Smoking is the most frequent preventable cause of cancer-related mortality, with infection, respiratory, hepatic, and renal failure the most frequent causes of death in cancer patients [3,4,5,6]. Cancer-related mortality decreased by 29% in 2017 compared to 1991 [6]. Owing to the advancement in screening, prevention, diagnosis, and therapeutics, the life expectancy and survival of cancer patients have increased significantly, which also increases the risk of developing additional primary malignancies in other organs [7,8].

Multiple primary malignancies (MPMs) are a frequently encountered phenomenon, with an incidence ranging from 0.52–11.7% [9,10,11]. This phenomenon does not include metastasis, and it is defined as the occurrence of two or more independent malignancies in the same or different organs [12,13]. MPMs are classified as synchronous or metachronous based on the time interval between malignancy diagnoses [11,14,15,16]. A synchronous malignancy is a second malignancy occurring either simultaneously, or within 6 months after the first malignancy, and a metachronous malignancy is a second malignancy at least 6 months after the first malignancy [11,14,15,16].

Although information regarding MPMs is not well-established, they can be a consequence of aging, chemoradiation for the first malignancy, an underlying genetic susceptibility, environmental exposures, immunosuppression, lifestyle factors, or hormonal factors [17]. Immunocompromised individuals might be at a higher risk of particular malignancies. Regardless, it may be true to hypothesize that some risk factors are shared by all patients with MPMs. For example, in kidney recipients, the rate of MPMs is estimated at 13.4%, which is higher than in the general population [18]. Inherited syndromic malignancies, such as familial adenomatous polyposis, Lynch syndrome, Li–Fraumeni syndrome, and Cowden syndrome, constitute a considerable portion of MPMs [19,20].

The prognosis of MPMs relies mainly on the type and stage. It is not clear whether having multiple malignancies alters the patients’ prognosis. Even though the samples in most of the literature were small, data suggest that having a short interval between malignancy diagnoses, defined as 60 months, is a poor prognostic factor. Additionally, the literature indicates that the rate and survival of metachronous malignancies are higher than those of synchronous malignancies [12,13,14,18]. As expected, younger patients have a better prognosis [9].

Since geographical differences in the MPM rate, survival patterns, and types have been described in other countries, it is worth assessing the rate of synchronous and metachronous malignancies, as well as survival and distribution, in male and female patients in Saudi Arabia. An in-depth evaluation of this phenomenon would provide a baseline for the careful monitoring of patients treated for a particular primary cancer and ensure the early detection and treatment of other primary cancers.

## 2. Method

### 2.1. Objective

The objective of this study was to evaluate the mortality rate and distribution of the malignancies based on gender and the diagnosis time interval, malignancy combinations, and survival patterns in Saudi patients with biopsy-proven MPMs and to determine the prevalence of MPMs in Saudi Arabia.

### 2.2. Study Design and Setting

This was a descriptive, single-center, retrospective study conducted at King Abdulaziz Medical City (KAMC), Department of Medical Oncology, Ministry of the National Guard-Health Affairs, Riyadh, Saudi Arabia. KAMC is an academic, government-funded tertiary hospital that combines clinical care, training, academics with research, and state-of-the-art medical technologies.

### 2.3. Inclusion and Exclusion

**Figure 1:**
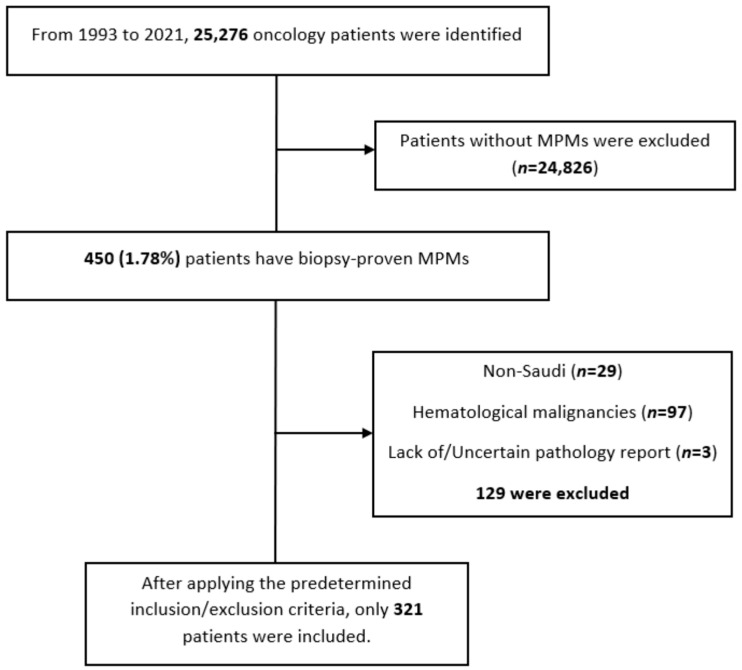
Shows a chart showing the details of the excluded patients. A total of 129 patients were excluded from the analysis, 29 of whom were non-Saudi, 97 with hematological malignancies, and 3 had no or uncertain pathology reports.

Initially, the Department of Medical Oncology tumor registry at KAMC identified a total of 25,276 oncology patients from 1993–2022. Then, 450 patients with at least two biopsy-proven malignancies were extracted. After that, the research team screened the 450 cases, and only 321 were eligible for inclusion. The included patients had to be Saudi with at least two biopsy-proven solid malignancies. Patients with hematological malignancies, such as leukemia, lymphoma, plasma cell dyscrasias, and myeloproliferative neoplasms, were excluded. The patients with missing or uncertain data, benign tumors, suspected malignancies, a lack of pathology reports, or indecisive pathology reports, were excluded Figure 1. Our department used ICD-10 classification to determine the tumors’ anatomical locations and ICD-O-3 for the tumors’ staging.

### 2.4. Data Collection

Data from 1993 to 2015 were collected from the data management system of the Department of Medical Oncology at King Abdullah Specialized Children’s Hospital, KAMC. Data from 2016 to 2022 were obtained by screening the electronic records (via the KAMC electronic system—BestCare; Seoul, Korea: ezCaretech Co.) of the cases that were identified by the tumor registry. The collected data included gender, first and second anatomical and histopathological diagnoses, the date of each diagnosis, date of last seen/last follow-up or date of death, and survival status. A synchronous malignancy was defined as a second malignancy that occurs either simultaneously, or within 6 months after the first malignancy diagnosis, and a metachronous malignancy was defined as a second malignancy at least 6 months after the first malignancy diagnosis.

### 2.5. Statistical Analysis

The Statistical Package for the Social Sciences (SPSS) version 22 (Armonk, NY, USA: IBM Corp.) was used for data analysis. The categorical variables were presented as frequency and percentage, and the numerical variables as mean ± standard deviation. The time interval between the first and second primary diagnoses was presented as median and percentiles (Q1, Q3). The prevalence of MPMs was calculated by dividing the number of MPM patients prior to applying the aforementioned exclusion criteria (450) over the total number of oncology patients (25,276), and then multiplying by 100 (1.78%). The survival times were calculated from the date of diagnosis of the first primary malignancy to the date of death or date of last follow-up. The survival probabilities for the synchronous and metachronous malignancies were estimated using the Kaplan–Meier method. The log-rank test was used to compare the survival times between different groups. Results were considered significant if *p* < 0.05.

### 2.6. Ethical Considerations

The study was approved by the Institutional Review Board of King Abdullah International Medical Research Center, Ministry of the National Guard-Health Affairs, Riyadh, Saudi Arabia (NRC22R/083/02). Because of the retrospective nature of the study, informed consent was waived. Access to the data was restricted to the researchers. The confidentiality of all patients was protected; no names or medical record numbers were used. Privacy and confidentiality were assured, and all the data, both hard and soft copies, were kept in a secure place within the National Guard-Health Affairs premises.

## 3. Results

**Figure 2:**
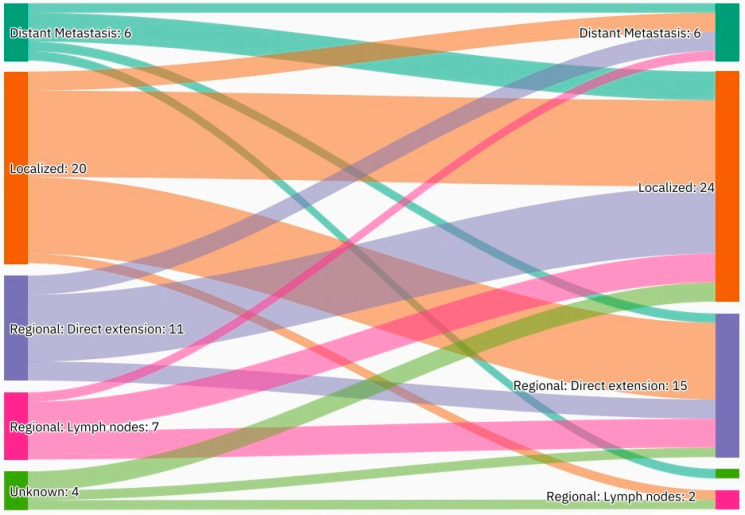
An alluvial plot showing stages of first and second primary malignancies. Detailed description: A total of 10.9% of the patients had their first primary malignancy diagnosis when it already metastasized to a distant organ, 21.8% had regional metastasis, and the rest had localized disease. A total of 16.8% of the patients had metastatic disease when diagnosed, 17.8% had regional metastasis, and the rest had localized disease.

**Figure 3:**
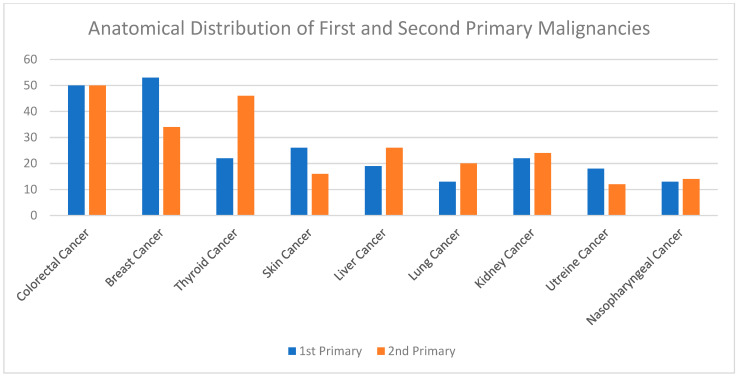
Shows the most frequent site of the first and second primary malignancies. Breast cancer was the most common first primary diagnosis, followed by colorectal and skin cancers. Colorectal cancer was the most frequent second primary diagnosis, followed by thyroid and breast cancers.

**Figure 4:**
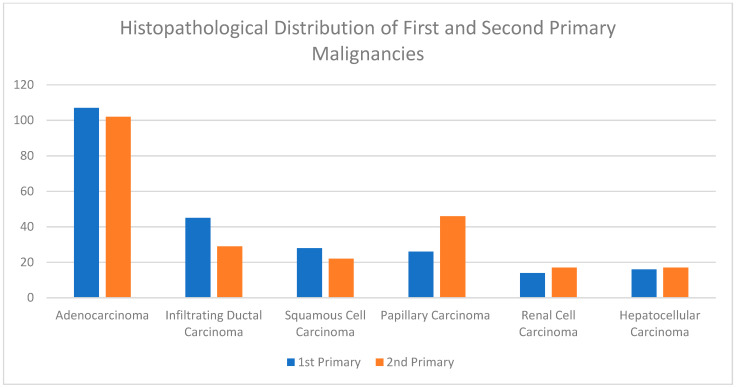
Shows the most frequent histopathologic morphology of the first and second primary malignancies. Adenocarcinoma was the most common first primary histopathology, followed by infiltrating ductal carcinoma and squamous cell carcinoma. Adenocarcinoma was also the most common second primary histopathology, followed by papillary carcinoma and ductal carcinoma.

**Figure 5:**
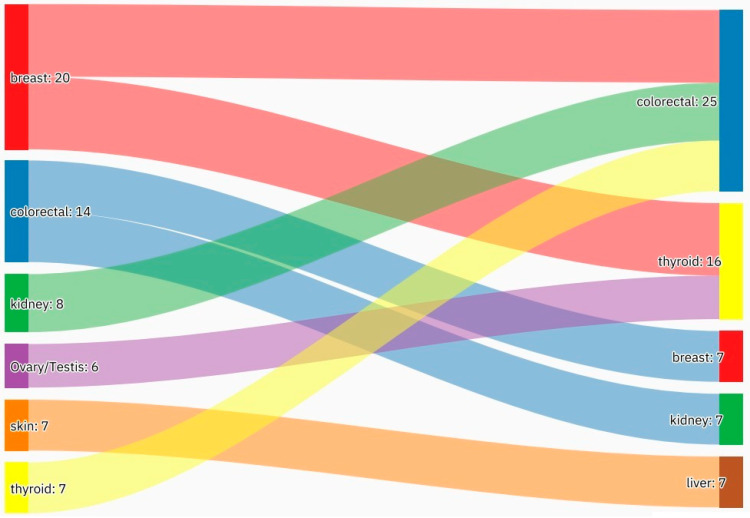
An alluvial plot showing the most frequent malignancy combinations. The most frequent malignancy combinations were breast–colorectal, breast–thyroid, kidney–colorectal, and colorectal–breast. Further frequent malignancy combinations are provided in Table 3.

**Figure 6:**
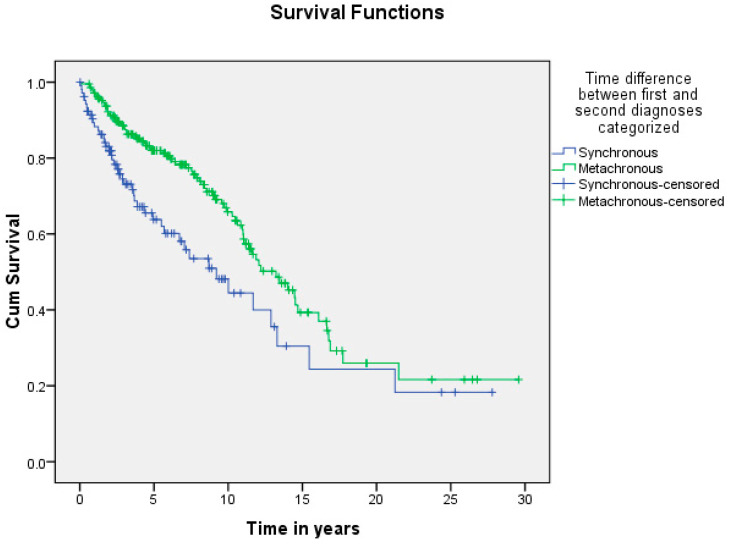
Shows the difference in survival of patients with synchronous and metachronous malignancies. The median survival time was 9.2 ± 1.7 and 13.2 ± 1.2 years for synchronous and metachronous malignancies, respectively, and the difference was statistically significant (*p* = 0.002).

**Figure 7:**
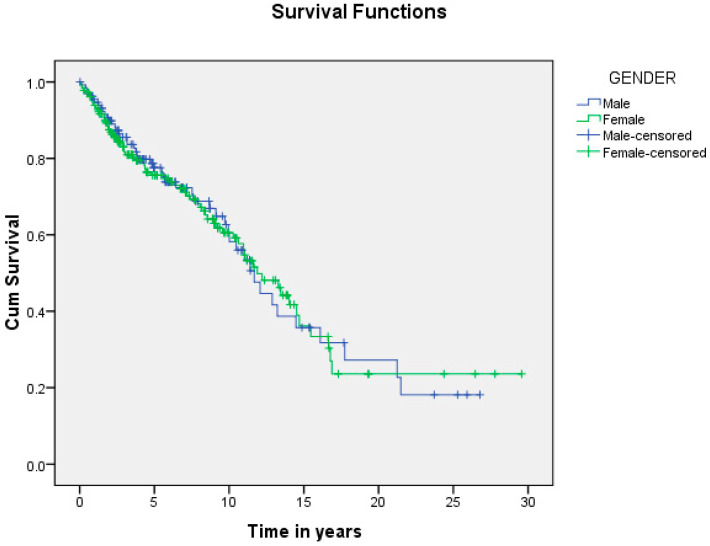
Shows the difference in survival based on patients’ gender. The median survival time in the males was 11.7 ± 1.0 years, and in the females, 11.9 ± 1.1 years, with no significant difference (*p* = 0.928).

**Figure 8:**
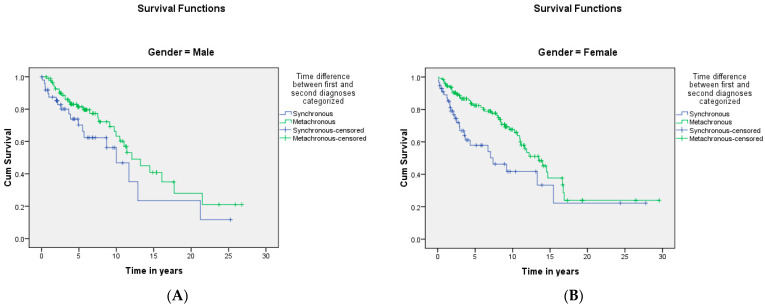
(**A**,**B**) shows the difference in survival when classified based on the MPM category by gender. The median survival time was 10 ± 1.7 and 12.1 ± 1.6 years for synchronous and metachronous malignancies, respectively, in the males, and in the females, the median survival time was 7.4 ± 2.6 and 13.5 ± 1.3 years for synchronous and metachronous malignancies, respectively. A significant difference was detected with the log-rank test (*p* = 0.002).

A summary of the results is provided in Table 1, Table 2 and Table 3. There was a total of 450 patients with MPMs from 1993-2022, with 321 eligible patients. More than half (57.3%; *n* = 184) of the patients were female. Only 4.0% (*n* = 13) of the cases had triple primary malignancies, with colorectal (*n* = 4) and appendiceal (*n* = 2) cancers being the most frequent third primary diagnoses. The other third primary diagnoses were gastric, liver, prostate, bladder, and cervical cancers (*n* = 1 for each). The prevalence rate of MPMs was 1.78%. The mean age at diagnosis of the first, second, and third primary malignancies was 59.3 ± 14.3, 62.7 ± 13.4, and 71.9 ± 6.5 years, respectively, with a median of 61 (Q1, Q3: 52, 69), 63 (Q1, Q3: 56, 72), and 74 (Q1, Q3: 64, 77), respectively. A third (33.0%; *n* = 106) were synchronous, and 66.7% (*n* = 214) were metachronous, with a median diagnostic interval of 1.72 (Q1, Q3: 0.15, 5.8) years. The overall mortality rate was 37.4% (Table 1). A small proportion (10.9%) had their first primary malignancy diagnosis when it already metastasized to a distant organ, 21.8% had regional metastasis, and the rest had localized disease. For the group with a second MPM, 16.8% had metastatic disease when diagnosed, 17.8% had regional metastasis, and the rest had localized disease (Figure 2). The most frequent site of the first primary malignancy was breast cancer (16.5%), followed by colorectal (15.6%), skin (8.1%), thyroid (6.9%), kidney (6.9%), and liver cancers (5.9%). Regarding the second primary malignancy, colorectal cancer (15.6%) was the most frequent, followed by thyroid (14.3%), breast (10.6%), liver (8.1%), and kidney (7.5%) cancers (Figure 3). The most frequent histopathologic morphology of the first primary malignancy was adenocarcinoma (33.3%), followed by infiltrating ductal carcinoma (14.0%), squamous cell carcinoma (8.7%), papillary carcinoma (8.1%), and hepatocellular carcinoma (5.0%). The most frequent histopathologic morphology of the second primary malignancy was adenocarcinoma (31.8%), followed by papillary carcinoma (14.3%), ductal carcinoma (9.0%), squamous cell carcinoma (6.9%), and renal cell and hepatocellular carcinoma (5.3% for both) (Figure 4). The most frequent synchronous malignancies were colorectal (*n* = 34), breast (*n* = 26), kidney (*n* = 24), thyroid, and liver (*n* = 22 for both) malignancies, and the most frequent metachronous malignancies were colorectal (*n* = 70), breast (*n* = 61), thyroid (*n* = 46), and skin (*n* = 33) malignancies. The most frequently observed histopathology for both synchronous and metachronous malignancies was adenocarcinoma (Table 2). The most frequent malignancy combinations (first primary malignancy–second primary malignancy) were breast–colorectal (*n* = 10), breast–thyroid (*n* = 10), kidney–colorectal (*n* = 8), colorectal–breast (*n* = 7), colorectal–kidney (*n* = 7), thyroid–colorectal (*n* = 7), skin–liver (*n* = 7), and ovary/testis–thyroid (*n* = 6) (Table 3 and Figure 5). The median survival time was 9.2 ± 1.7 and 13.2 ± 1.2 years for synchronous and metachronous malignancies, respectively, and the difference was statistically significant (*p* = 0.002) (Figure 6). The median survival time in the males was 11.7 ± 1.0 years, and in the females, it was 11.9 ± 1.1 years, with no significant difference (*p* = 0.928) (Figure 7). If classified according to the MPM category by gender, the median survival time was 10 ± 1.7 and 12.1 ± 1.6 years for synchronous and metachronous malignancies, respectively, in the males, and in the females, the median survival time was 7.4 ± 2.6 and 13.5 ± 1.3 years for synchronous and metachronous malignancies, respectively. A significant difference was detected with the log-rank test (*p* = 0.002) (Figure 8A,B).

## 4. Discussion

Cancer-related mortality decreased by 29% in 2017 compared to 1991, reflecting significant achievements in cancer screening, prevention, diagnosis, and treatment, [6,21] which has increased patients’ overall survival. Keeping the multistep clonal origin of cancer theory in mind, cancer develops as a consequence of somatic changes in the DNA, resulting in unregulated cellular proliferation [22]. These changes, in the form of subtle mutations, might be the result of random errors during replication or of carcinogen exposure. Based on this, the increase in patients’ survival exposes them to more mutations and epigenetic changes, increasing their risk of developing another primary malignancy. Since the early 1970s, cancer patients have had 1.29 times the risk of developing another primary malignancy compared to the general population [23].

Although information regarding MPMs is limited, several risk factors have been identified, including aging, genetic susceptibility, chemotherapy such as doxorubicin and cyclophosphamide, radiation, immunosuppression, and smoking [17,18,24,25]. Individuals diagnosed with cancer at a relatively young age are at an increased risk of MPMs due to the increased exposure to risk factors and more aggressive treatment. The purpose of this study was to create a baseline supporting the monitoring of patients treated for a particular primary malignancy in order to ensure the early detection of another primary malignancy by determining the distribution of the malignancies based on gender and time interval, type of paired malignancies, and the survival patterns of both synchronous and metachronous malignancies in Saudi Arabia.

In the current study, MPMs were more prevalent in the females (57.3%) than in the male patients (42.7%), which is not supported by the literature [9,11,12,26]. The higher rate of MPMs seen in male patients could be explained by drinking alcohol and smoking tobacco. Although smoking is prevalent in Saudi Arabia, alcohol is illegal and rarely used due to religious and cultural reasons. This could partially justify the female predominance in our study. Another explanation could be the fact that many syndromic cancers, such as Lynch and Li-Fraumeni syndromes, involve gynecologic malignancies, and with the high rate of consanguinity in our population (66.7%), females would be at a higher risk of MPMs [27].

Only 4.0% of our sample had triple primary malignancies, in agreement with the literature [26,28]. The literature reported colorectal, intracranial, and gynecologic malignancies as the most frequent third primary diagnoses; however, the current study indicated colorectal and appendiceal malignancies as the most frequent [26,29]. This supports the geographical differences in the patterns of MPMs. We did not find cases with four primary malignancies, although it is reported in the literature [12,30,31].

Our results showed that 33.0% of the cases were synchronous, with 67.0% being metachronous malignancies. This pattern is consistent with the literature, as synchronous MPMs range from 30.0–55.0% [9,32,33]. This could be explained by geographical differences, hospital diagnostic tools, and the characteristics of the patients. Another explanation may be related to patient compliance, as evidenced in the current study, with 10.9% of the first primary malignancies already metastasized at the time of diagnosis, and 21.8% metastasized to the regional lymph nodes. However, 16.8% of the second primary malignancies were also metastatic to distant organs at the time of diagnosis, and 17.8% were metastatic to regional lymph nodes. The higher proportion of distant metastasis of the second primary malignancy at the time of diagnosis may reflect poor patient compliance to regular follow-ups and surveillance, as the second malignancy may develop within the first 6 months of the first malignancy, but not be detected. The importance of compliance with follow-ups and surveillance should be explained to the patients, and a referral to mental health or counselling should be considered if needed. The overall rate of MPMs ranges from 0.52–17.0%, depending on the definition, although we were not able to calculate it for the Saudi population [9,10,11,34,35,36].

In the current study, breast cancer was the most frequently diagnosed first primary malignancy, followed by colorectal, skin, thyroid, kidney, and liver cancers, accounting for 16.5%, 15.6%, 8.1%, 6.9%, 6.9%, and 5.9%, respectively. This is probably unique to the Saudi population, as definite geographical differences exist in different populations and countries. To emphasize, based on the results of two Turkish studies, the most frequent first primary malignancies were head and neck, breast, colorectal, bladder, and gastric cancers [26,37]. In Indian patients, head and neck, breast, colorectal, gynecologic, and soft tissue cancers were the most commonly diagnosed first primary malignancies [37].

In China, the most frequently diagnosed first primary metachronous malignancies were colorectal, head and neck, and lung cancers, and the most frequently diagnosed first primary synchronous malignancies were lung, colorectal, and breast cancers [9,12,38]. Our results showed that colorectal, breast, kidney, thyroid, and liver malignancies were the most frequent synchronous malignancies, with colorectal, breast, thyroid, and skin malignancies being the most frequent metachronous malignancies. In support, in our study, colorectal cancer was the most frequent second primary malignancy, followed by thyroid, breast, liver, and kidney cancers. However, in Turkey, lung cancer was the most frequent, followed by colorectal, breast, and gynecologic cancers [26,37]. In China, colorectal cancer was the most frequent, followed by lung and breast cancers [9,12,38,39]. These findings indicate clear differences based on the country in which the study was conducted.

In our study, the most frequently observed histopathologic morphology in synchronous and metachronous malignancies was adenocarcinoma. The histopathologic morphology in patients with MPMs is not always reported in the literature. A Chinese study reported that adenocarcinoma was the most frequent histopathological diagnosis in both the synchronous and metachronous groups, followed by squamous cell carcinoma [9,40].

In the current study, the most frequently observed malignancy pairs (first primary malignancy–second primary malignancy) were breast–colorectal, breast–thyroid, kidney–colorectal, colorectal–breast, colorectal–kidney, thyroid–colorectal, and skin–liver. Based on a Turkish study, the most frequently observed malignancy pairs in males were head and neck–lung, bladder–lung, bladder–prostate, and prostate–lung. For females, the most frequently observed malignancy pairs were breast–gynecologic, colorectal–breast, breast–colorectal, gynecologic–breast, and mesenchymal malignancy combinations [26]. Although we did not study the distribution of the malignancies in the males and females separately, the results are contrasting. A European study showed that the most frequent malignancy combinations were colorectal–urogenital (urinary bladder and prostate) in males and colorectal–gynecologic in females, [41] and in Jordan, the most frequently observed pairs were colorectal–skin, colorectal–kidney, kidney–skin, and lung–lung [28]. Finally, in India, a study with 38 patients with MPMs showed that head and neck–head and neck was the most frequently observed malignancy combination [37]. It is crucial to keep in mind that these differences might also depend on the lifespan and accessibility of health services to the general population.

In this study, the median survival time was 9.2 and 13.2 years for synchronous and metachronous malignancies, respectively, and the difference was statistically significant (*p* = 0.002). This is in agreement with the literature reporting worse survival with synchronous malignancies [12,42]. A possible reason is the fluctuation of the immune system. To clarify, initially, malignancy weakens the immune system, but subsequently triggers the immune system via cytokines and the release of growth factors, eventually resulting in an exhausted and weak immune system. There is a possibility that the strengthened immunity is the cause of the longer survival in patients with metachronous MPMs. However, in patients with synchronous MPMs, due to the short interval, the immune system is seriously compromised and cannot be triggered and strengthened, compromising survival [12,43]. The differences in the survival patterns between the males and females were not significant, which is consistent with the literature [12].

The current findings indicate geographical differences in different countries. Although the number of people surviving cancer is increasing, the burden of cancer will continue to grow as the likelihood for subsequent primary cancers increases. An important contribution of the current findings is raising awareness among oncologists, with increased attention and suspicion of particular malignancies that are more likely to coexist synchronously or metachronously. In terms of future research, it would be useful to extend the current findings by examining the association in these paired malignancies, the complications, and therapeutic implications in order to improve the findings of this descriptive and single-center study. Despite these limitations, the present study enhanced our understanding of the probability of the coexistence of particular malignancies in Saudi Arabia. We hope that the current research will encourage future researchers to investigate this important area.

## 5. Conclusions

The most frequent site of the first primary malignancy was breast cancer, followed by colorectal, skin, and thyroid cancers. The most frequent site of the second primary malignancy was colorectal cancer, followed by thyroid, breast, and liver cancers. Only 4.0% of the cases had a third primary malignancy, with colorectal and appendiceal cancers being the most frequent third primary diagnoses. The most frequent malignancy combinations were breast–colorectal, breast–thyroid, kidney–colorectal, colorectal–breast, colorectal–kidney, thyroid–colorectal, and skin–liver. The synchronous malignancies had a lower survival time. The possibility of developing another primary malignancy should be considered when treating and monitoring cancer patients.

## Figures and Tables

**Table 1:** General Information regarding Multiple Primary Malignancies.

	*n*	%
**Gender:**		
Male	137	42.7
Female	184	57.3
**Number of Multiple Primary Malignancies:**		
>2	13	4.0
**Distribution of Malignancies Based on the Time Interval between 1st and 2nd Diagnosis:**		
Synchronous Malignancies	106	33.0
Metachronous Malignancies	214	66.7
Survival Status:		
Alive	201	62.6
Dead	120	37.4
**^a^** **Distribution of Mortality Based on the Time Interval between 1st and 2nd Diagnosis:**		
Synchronous Malignancy Mortality	44	41.5
Metachronous Malignancy Mortality	76	35.5
**^a^** **Distribution of Mortality Based on the Patients’ Gender:**		
Male Mortality	49	35.8
Female Mortality	71	38.6

^a^ No significance was found based on chi-square test.

**Table 2:** Anatomical Distribution of Multiple Primary Malignancies and the Histopathology Based on the Time Interval between the 1st and 2nd Diagnosis.

	Synchronous Malignancies *n*				Metachronous Malignancies *n*			
**Anatomical** **Location:**	**First**	**Second**	**Third**	**Total**	**First**	**Second**	**Third**	**Total**
Colorectal	16	17	1	34	34	33	3	70
Breast	11	15	0	26	42	19	0	61
Thyroid	8	14	0	22	14	32	0	46
Skin	7	3	0	10	19	13	1	33
Lung	8	3	0	11	5	17	1	23
Uterine	7	2	0	9	11	10	0	21
Gastric	3	3	1	7	2	9	0	11
Liver	11	10	1	22	8	16	0	24
Kidney	5	19	0	24	17	5	0	22
Pancreas	4	0	0	4	6	8	0	14
Prostate	5	4	0	9	6	8	1	15
Nasopharynx/Larynx	4	6	0	10	9	8	0	17
Ovary/Testis	6	3	0	9	11	4	0	15
Bone and Soft Tissue	1	1	0	2	3	5	0	8
Small Intestine	2	1	0	3	1	2	0	3
Gallbladder/ Ductal System	2	0	0	2	5	3	0	8
Urinary Bladder	4	3	0	7	8	6	1	15
Vagina/Cervix	1	0	0	1	6	4	1	11
Brain and Eye	0	0	0	0	7	4	0	11
Appendix	1	2	0	3	0	3	2	5
Unknown Primary Origin	0	0	0	0	1	2	0	3
	**Synchronous Malignancies *n***				**Metachronous Malignancies *n***			
**Histopathology:**	**First**	**Second**	**Third**	**Total**	**First**	**Second**	**Third**	**Total**
Adenocarcinoma	42	23	1	66	65	79	6	150
Squamous Cell Carcinoma	8	6	0	14	20	16	1	37
Papillary Carcinoma	10	12	0	22	16	34	0	50
Melanoma	1	0	0	1	1	3	0	4
Basal Cell Carcinoma	1	2	0	3	9	6	0	15
Sarcoma	3	3	1	7	3	12	0	15
Renal Cell Carcinoma	1	14	0	15	13	3	0	16
Hepatocellular Carcinoma	9	8	1	18	7	9	0	16
IDC	9	12	0	21	36	17	0	53
ILC	1	1	0	2	1	0	0	1
Adenosquamous	1	0	0	1	1	2	0	3
Translational Cell	3	7	0	10	11	3	1	15
Neuroendocrine	0	1	0	1	1	1	0	2
Teratoma/ Mixed Cell	2	0	0	2	1	0	0	1
Small-Cell Carcinoma	1	0	0	1	0	6	0	6
Carcinoma NOS/Others	14	16	0	30	26	21	2	49

Abbreviations. IDC: Infiltrating/Invasive Ductal Carcinoma; ILC: Infiltrating/Invasive Lobular Carcinoma; NOS: Not Otherwise Specified.

**Table 3:** The Most Frequent Malignancy Combinations.

First Malignancy:	Second Malignancy:	*n*	% ^a^
Breast Cancer	Colorectal Cancer	10	18.9
Breast Cancer	Thyroid Cancer	10	18.9
Kidney Cancer	Colorectal Cancer	8	36.4
Colorectal Cancer	Breast Cancer	7	14.3
Colorectal Cancer	Kidney Cancer	7	14.3
Thyroid Cancer	Colorectal Cancer	7	31.8
Skin Cancer	Liver Cancer	7	26.9
Ovarian/Testicular Cancer	Thyroid Cancer	6	35.3
Liver Cancer	Kidney Cancer	5	26.3
Endometrial Cancer	Colorectal Cancer	5	27.8
Lung Cancer	Thyroid Cancer	5	38.5
Urinary Bladder Cancer	Lung Cancer	5	41.7
Colorectal Cancer	Lung Cancer	5	10.2
Colorectal Cancer	Skin Cancer	5	10.2
Colorectal Cancer	Thyroid Cancer	5	10.2
Breast Cancer	Liver Cancer	5	9.4
Breast Cancer	Endometrial Cancer	5	9.4

^a.^ These percentages represent the percentages of the second malignancy among the first malignancy only, not the whole sample.

## Data Availability

The data presented in this study is available on request from the corresponding author.
